# Pharmacological Therapy in the Heart as an Alternative to Cellular Therapy: A Place for the Brain Natriuretic Peptide?

**DOI:** 10.1155/2016/5961342

**Published:** 2016-01-04

**Authors:** Nathalie Rosenblatt-Velin, Suzanne Badoux, Lucas Liaudet

**Affiliations:** ^1^Division de Physiopathologie Clinique, Centre Hospitalier Universitaire Vaudois and University of Lausanne, 1005 Lausanne, Switzerland; ^2^Service de Médecine Intensive Adulte, Centre Hospitalier Universitaire Vaudois and University of Lausanne, 1005 Lausanne, Switzerland

## Abstract

The discovery that stem cells isolated from different organs have the ability to differentiate into mature beating cardiomyocytes has fostered considerable interest in developing cellular regenerative therapies to treat cardiac diseases associated with the loss of viable myocardium. Clinical studies evaluating the potential of stem cells (from heart, blood, bone marrow, skeletal muscle, and fat) to regenerate the myocardium and improve its functional status indicated that although the method appeared generally safe, its overall efficacy has remained modest. Several issues raised by these studies were notably related to the nature and number of injected cells, as well as the route and timing of their administration, to cite only a few. Besides the direct administration of cardiac precursor cells, a distinct approach to cardiac regeneration could be based upon the stimulation of the heart's natural ability to regenerate, using pharmacological approaches. Indeed, differentiation and/or proliferation of cardiac precursor cells is controlled by various endogenous mediators, such as growth factors and cytokines, which could thus be used as pharmacological agents to promote regeneration. To illustrate such approach, we present recent results showing that the exogenous administration of the natriuretic peptide BNP triggers “endogenous” cardiac regeneration, following experimental myocardial infarction.

## 1. Introduction

Cardiovascular diseases (CVDs) account for 30% of all deaths worldwide, which represented 17.3 million fatalities in 2008 (World Health Organization, Fact sheet number 317), among which 13.5 million (80%) were related to the consequences of coronary heart diseases (CHDs). This number is expected to rise steadily, with an estimated 23.3 million deaths in 2030. The identified causes of this “epidemics” involve a sedentary life of style, an unhealthy diet, as well as the use of tobacco and/or alcohol consumption [1, 2]. All favor the emergence of obesity, diabetes, and/or hypertension which are risk factors for CHDs.

Many efficient therapies have been developed to treat CVDs over the past 30 years, including various reperfusion strategies of occluded coronary vessels, antiplatelet and anticoagulant agents to prevent/treat coronary thrombosis, beta-blocking drugs, or angiotensin-converting enzyme inhibitors, to name only a few [3]. However, despite the identification of risk factors and the improvements in therapy, the morbidity and mortality associated with CHDs remain unacceptably high. A major reason for it is that CHDs induce the loss of a given amount of contractile myocardium, with unavoidable consequences on the functional activity of the heart. Indeed, the mammalian heart has long been considered a postmitotic organ with no capacity to regenerate [4], which is in striking contrast with certain lower vertebrates (zebrafish, urodeles), which have a high cardiac regeneration rate. The various treatments aimed to delay the onset of heart failure or to limit the consequences of CVDs, do not have the ability to replace the damaged cardiac cells, especially the necrotic and/or apoptotic cardiomyocytes [5], and thus cannot properly “heal” the injured heart. This view has begun to change dramatically with the discovery that the adult heart displays some capacity to regenerate after damage and, hence, that manipulating such regenerative capacity might have therapeutic potential. These emerging concepts will be here concisely reviewed.

## 2. Regenerative Capacities of the Adult Mammalian Heart

In the last decade, intensive research in the cardiovascular field has allowed a more precise understanding of the cellular and molecular mechanisms governing cardiomyocyte differentiation and proliferation during physiological growth, ageing, and pathophysiological conditions. A milestone observation was the demonstration that cardiac regeneration represents a physiological process occurring during ageing in normal conditions [6]. Although the proportion of newly formed cardiomyocytes is currently debated, the fact that new cardiomyocytes are generated in human hearts during physiological ageing and after heart injuries is now well admitted [6–8]. Different mechanisms have been identified to account for the* de novo* generation of cardiomyocytes in the adult heart. These mechanisms, detailed below, include the proliferation of the preexisting mature cardiomyocytes with or without dedifferentiation, the differentiation of endogenous precursor cells, and the differentiation of exogenous infiltrating cells (for review see [9]).

### 2.1. Proliferation of Mature Cardiomyocytes

Although cardiomyocytes in mammals demonstrate proliferative capacities during fetal development, it has been commonly admitted that after birth, cardiomyocytes cannot reenter the cell cycle, as DNA replication occurs without cytokinesis or karyokinesis [10]. This assumption was first challenged by the Sadek laboratory, who demonstrated that mouse cardiomyocytes can proliferate after partial surgical resection of the heart at birth [11]. In this mouse model, cardiomyocyte proliferation led to the replacement of the resected tissue and the inhibition of fibrosis. Notwithstanding this obvious regenerative process, the capacity of murine cardiomyocytes to proliferate was lost after 7 days of age. Further evidence of cardiomyocyte ability to proliferate came from the Lee laboratory, who recently proposed that preexisting cardiomyocytes represent the main source of newly formed cardiomyocytes during ageing, as well as following myocardial infarction (MI) [12]. However, although cardiomyocyte proliferation occurs life-long, this process is seldom in the mouse heart after the first month of life [13].

The mechanisms by which cardiomyocytes are able to proliferate are not well established. In zebrafish hearts, mature cardiomyocytes have to dedifferentiate before proliferating [14]. During this dedifferentiation, cardiomyocytes reduce their sarcomere structure (they become smaller and round) and reexpress the alpha skeletal actin (*α*-ska) protein as well as cardiac progenitor cell markers, such as Nkx2.5 and c-kit. They downregulate the expression of prototypical markers of mature cardiomyocytes, such as Troponin I and *α*-myosin heavy chain (*α*-MHC). Their new structure and phenotype facilitate their reentry into the cell cycle. This process has also been observed* in vitro* in cardiomyocyte isolated from rat hearts [15]. However, whether this process occurs* in vivo* in mammal hearts is under debate. Dedifferentiated cardiomyocytes have been detected in the hearts of infarcted sheep hearts or in pressure-and-volume overloaded rabbit hearts [16, 17]. In human hearts after idiopathic dilated cardiomyopathy, infarction or atrial fibrillation dedifferentiated cardiomyocytes were also detected [18, 19]. The presence of these cells has been shown to be dependant at least in part by Oncostatin M [20]. However the results published until now did not demonstrate a direct link between the cardiomyocyte dedifferentiation and the proliferation. In other words, the results demonstrating that dedifferentiated cardiomyocytes proliferate* in vivo* are lacking.

However, stimulation of the cardiomyocyte proliferation appears as a new therapeutical strategy to increase cardiac regeneration especially in pathophysiological conditions. Several factors have been identified to be able to induce cardiomyocytes to reenter the cell cycle: Neuregulin 1 and its ERBB2 receptor [21–24], Periostin [25], the fibroblast growth factor-1 [26, 27], or also the stromal cell-derived factor 1*α* [28]. The use of miRNAs is also investigated and demonstrated that hsa-miR-590 and hsa-miR-199a were able to stimulate cardiomyocyte proliferation [29]. Interestingly, new results published by Sadek laboratory demonstrated that hypoxia is a crucial factor able to stimulate cardiomyocyte proliferation [30]. The authors identified in adult mouse hearts a small population of proliferating cardiomyocytes expressing Hif-1*α* and able to give rise to new cardiomyocytes (at a rate of 0.3–1% per year) during physiological ageing. Thus, these results could explain why in neonatal hearts (relatively more hypoxic than adult hearts) cardiomyocytes proliferate. Thus, the oxygen postnatal environment which has been shown to lead to DNA damage response [31], appears as a major regulator of cardiomyocyte proliferation. However, the regulation of other genes such as p21 or the transcription factor Meis1 [32, 33] or the mechanical loading of the hearts [34] could also contribute to the arrest of cardiomyocyte proliferation in postnatal hearts.

### 2.2. Differentiation of Endogenous Precursor Cells

**Figure 1:**
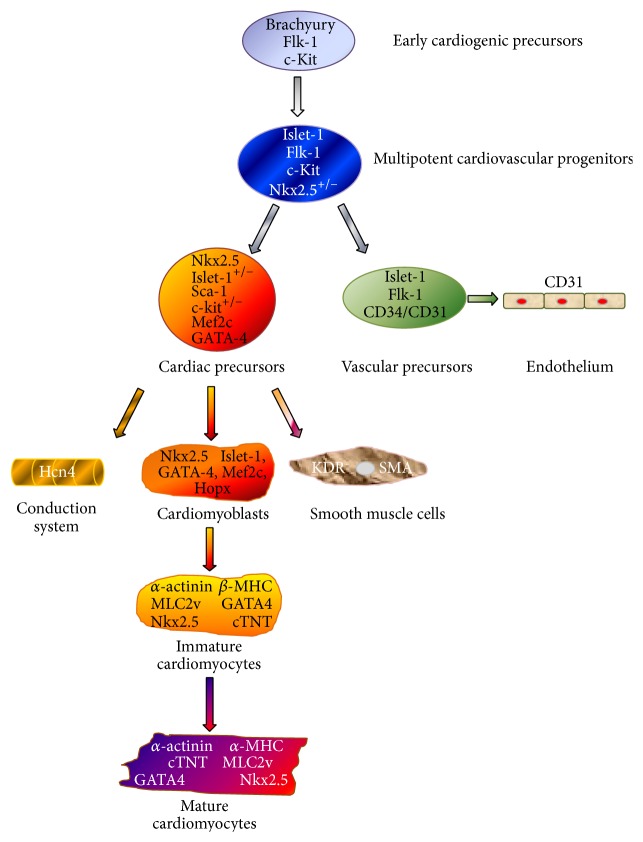
Cardiovascular cell lineage. Schematic representation depicting the origin of cardiomyocytes and endothelial and smooth muscle cells, as well as the conduction system. Several proteins are associated with the different stage of differentiation of the cardiac cells: islet-1, Flk-1, and c-kit are expressed at an undifferentiated stage, whereas the expression of Nkx2.5 and Sca-1 identifies more differentiated cardiac precursor cells.

Cardiac precursor cells (CPCs), which have the capacity to differentiate into mature functional cardiomyocytes, exist in the heart itself. The characterization of these cells remains a difficult task, due to the lack of a defined, highly specific marker. Thus the association of several markers is required to identify cardiovascular progenitor and cardiac precursor cells [35, 36] (Figure 1). Early cardiogenic precursors originate from mesoderm and are identified as expressing the c-kit protein [37–39] (a cellular cytokine receptor initially found at the surface of hematopoietic progenitor cells), the vascular endothelial growth factor (VEGF) receptor 2 protein (Flk-1) [40], and the nuclear transcription factor islet-1 [35, 41, 42]. These relative undifferentiated cells give rise to multipotent cardiovascular progenitors which express the nuclear transcription factor Nkx2.5 [43, 44] together with the islet-1, Flk-1, and c-kit proteins. These progenitors differentiate into vascular precursors expressing the endothelial markers CD34 and CD31 or into cardiac precursor cells expressing notably Nkx2.5, GATA-4, Mef2c, and the stem cell antigen-1 (Sca-1) proteins [42, 45–47]. CPCs can differentiate into cells of the conduction system, into smooth muscle cells, and into cardiomyoblasts expressing Hopx [48]. Hopx^+^ cells give only rise to cardiac myocytes (immature and mature cardiomyocytes).

The participation of the endogenous CPCs to heart regeneration in physiological conditions is controversial [8, 12]. Under pathological conditions, it is now well established that CPCs can differentiate into cardiomyocytes when they were activated with different stimuli, such as FGF-2, thymosin *β*4, prostaglandin E2, human stem cell factor, or also stromal-cell derived factor 1 (SDF1) [8, 49–53]. However, such involvement seems to be limited in time, as indicated by Hsueh and coworkers who reported that CPC differentiation into cardiomyocytes started at day 7 after MI but saturated on day 10 [51]. Interestingly, in senescent heart, CPCs are quiescent because of lack of stimulation but they can be re-activated by stem cell factor [39]. This suggests that, even in old hearts, activation of endogenous CPCs could be used as a therapeutical way to increase cardiac regeneration.

Among the “direct” activation of CPCs with several factors, the microenvironment of the CPCs can also be modified to increase their potency to participate into heart regeneration. Thus, their migration capacity which is dependent on the SDF1 secreted by the damaged myocardium and its CXCR 4 receptor (expressed by CPCs) can be modulated [54]. The group of Wang induced overexpression of SDF1 by the cardiomyocytes, which led to increased mobilization of CPCs [55]. SDF1 has also been shown to activate the endogenous cardioblasts in adult hearts after myocardial infarction [53].

### 2.3. Role of Infiltrating Cells from Extracardiac Origin

Although the role of infiltrating cells is not yet well defined, inhibition of certain aspects of inflammation is detrimental to cardiac repair after myocardial infarction [56–58], pointing to some role of infiltrating cells in the regenerative process. In this respect, evidence has accumulated that monocytes/macrophages are key players in this scenario [57], a concept notably supported by the increased mortality of MI in mice following transient macrophage depletion [58]. Two different subsets of monocytes originating from the bone marrow, with different, yet complementary functions, are mobilized in the heart after MI: the CD11b^high^/Ly6C^high^ subset infiltrates the heart 1–3 days after MI, exhibits phagocytic, proteolytic, and inflammatory functions, and represents 75% of the monocytes in the infarcted hearts at this stage (of note, Ly6C^high^ monocytes originating from the spleen have also be detected in the MI site [59]); the CD11b^high^/Ly-6C^low^ subset colonizes the heart from day 4 to day 7 and produces less inflammatory mediators but expresses vascular-endothelial growth factor (VEGF), thus promoting angiogenesis [56].

Thus whether a paracrine effect of these cells is now evident (for review see [60]), their ability to differentiate into mature cardiomyocytes remains controversial. Indeed, the differentiation into cardiomyocytes of cells isolated from the bone marrow (BMCs) or the blood was first demonstrated [61–65] and then challenged, with the suggestion that these cells might rather fuse with the native cells instead of differentiating [64, 66, 67]. Finally, now several reports demonstrated that both processes, actual differentiation and fusion, coexist [68, 69]. This was, for example, demonstrated for human circulating CD14^+^ monocytes infiltrating the infarcted myocardium [70] and recently for hematopoietic cells which are able to “fuse” with cardiomyocytes and/or “transdifferentiate” into cardiomyocytes. Whatever the fate of the circulating cells in the heart, numerous factors secreted by these cells have been identified, such as vascular endothelial growth factor (VEGF), insulin growth factor (IGF-1), growth hormone (GH), or hepatocyte growth factor (HGF). These factors promote angiogenesis and atherogenesis but can also stimulate endogenous CPC proliferation and differentiation [71].

## 3. Therapeutic Issues in Cardiac Regeneration

### 3.1. Cardiac Cell Therapies

The idea leading to the development of cellular therapy in damaged heart is to replace the large amount of cardiomyocytes which died after heart injuries. Thus, cellular therapies, consisting in injecting “cardiomyocyte precursor cells” from various sources into the injured hearts, have been evaluated as the first option in this novel therapeutic paradigm.

Three categories of stem cells could be used: embryonic, adult, and induced pluripotent stem cells (iPSCs). It is important to mention here that, due primarily to ethical issues, only one clinical trial performed so far has used cardiac progenitors derived from human embryonic cells (hESCs) [72]. However, the use of these cells is promising as they regenerate nonhuman primate hearts [73]. In the same way, iPSCs [74] were not yet tested in patients. Furthermore, the discussion is open concerning the use of stem cells from umbilical cord stroma [75].

**Figure 2:**
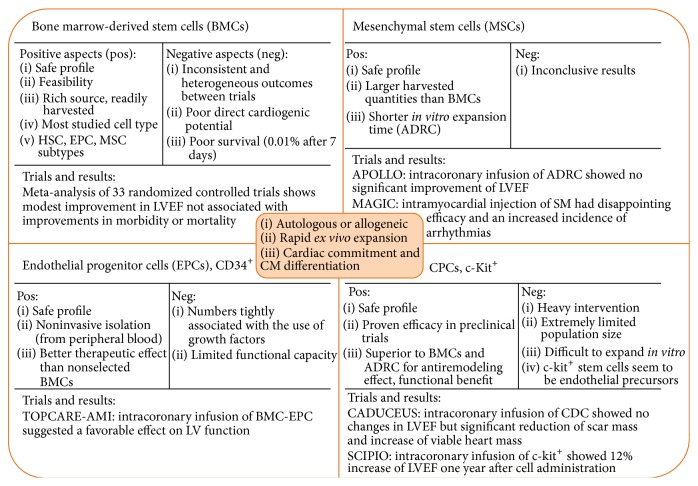
Current state of stem cell therapy for acute myocardial infarction in clinical trials. The optimal cell population for cardiac regenerative cell therapies requires autologous or allogeneic origin, rapid* ex vivo *expansion, and cardiac commitment including differentiation to cardiomyocytes. Numerous clinical trials have been undertaken with moderate results (for reviews see [8, 77, 172–174]). ADRC: adipose-derived stem cells; BMC: bone marrow cells; CDC: cardiosphere-derived cells; CM: cardiomyocytes; EPC: endothelial progenitor cells; HSC: hematopoietic stem cells; LVEF: left ventricular ejection fraction; MSC: mesenchymal stem cells; SC: stem cells.

Thus almost all clinical trials were performed with adult stem cells (Figure 2). The choice of the type of adult precursor cells to inject must be based on 3 main criteria. (1) They should be easily isolated from patients (“autologous cells”) or from healthy donors (“allogeneic cells”). (2) They should be expandable in large number (>100 millions in the case of bone marrow cells), implying that the cells should be kept in an undifferentiated state* in vitro*, to allow high proliferative capacity. (3) They should have the ability to differentiate into mature cardiomyocytes.

Two types of cells fulfilling these criteria have been used in clinical trials: cells isolated from the bone marrow, blood, skeletal muscle, or fat, referred to as “exogenous” precursor cells, and cells isolated from the heart itself (from atrial biopsies) referred to as “endogenous” precursor cells.

#### 3.1.1. The “Exogenous” Precursor Cells

The easiest precursor cells to isolate are obtained from the blood or the bone marrow (BMCs). Thus, a vast majority of clinical trials performed so far used BMCs, either unselected, or sorted according to some markers of undifferentiated BMCs (CD133^+^ or CD34^+^ enriched BMCs). Mesenchymal stem cells (MSCs), obtained by specific culture processing of the BMCs, have been frequently used as well and indeed are generally presented as the “most effective cells” which can be injected [76].

The results obtained using BMC injection (usually via an intracoronary or a percutaneous transendocardial injection) have been generally disappointing, as summarized in recent extensive reviews [76, 77]. A meta-analysis of 13 randomized trials of unsorted BMC injection in patients with acute MI concluded that BMCs did not prevent the remodeling process [78]. The REPAIR-AMI trial, which is a multicenter double-blind trial of the intracoronary injection of BMCs after acute MI, reported a 5.5% increase of left ventricular ejection fraction in post-MI patients at 6 months [79]. However, 18 months after cell injection, no significant difference in left ventricular ejection fraction was detected between cell and placebo injected patients included in the REPAIR-AMI trial. Similarly, BMC injection in patients after ST-elevation myocardial infarction (BOOST trial) led to 6% increase of the left ventricular ejection fraction 6 months after cell injection (*P* = 0.003) but only to 2.8% at 18 months (*P* = 0.27) [80]. Recently a meta-analysis using the individual data of the patients involved in 12 randomized trials concluded that intracoronary injection of bone marrow cells after MI provides no benefit for the patients [81].

However, with respect to CD133^+^ enriched BMCs and MSCs, their administration after acute MI did result in moderate improvements of cardiac parameters when compared to control patients with in some cases a small reduction of the absolute scar size [82–84]. Furthermore, patients injected with a larger percentage of CD31^+^ cells among their BMCs, demonstrated a greater reduction of infarct size than patients injected with smaller percentage of CD31^+^ [85]. This clearly demonstrates that the nature of the cells which are injected is crucial for the outcome of the therapy.

Cells other than BMCs have been used in some clinical trials. In the MAGIC trial, patients undergoing coronary bypass surgery for previous MI and severe left ventricular dysfunction were injected with skeletal myoblasts (cultured from a muscle biopsy) within the myocardial scar. Myoblast transfer did not improve LV function in comparison to control patients and was associated with early postoperative arrhythmias [86]. Finally, two recent clinical studies used adipose tissue-derived regenerative cells (ADRCs, isolated from liposuction aspirates), administered to patients with acute MI [87] or severe chronic ischemic cardiomyopathy [88]. Results of these studies are encouraging, as ADRCs were associated with a 50% reduction of myocardial scar formation post-MI and a preserved ventricular function in patients with ischemic cardiomyopathy. Additional studies are needed to confirm these preliminary results.

#### 3.1.2. The “Endogenous” Precursor Cells

Precursor cells do exist within the heart, but their identification has been made difficult by the lack of a highly specific marker. Cells expressing the c-kit have been isolated from the heart, induced to proliferate* in vitro* and reinjected into patients as cardiac precursor cells from “autologous cardiac origin.” The first clinical trials with autologous CPCs used c-kit^+^ cells obtained from atrial biopsies (SCIPIO study) or from cardiospheres (self-assembling multicellular clusters containing various progenitor cells) obtained from right ventricular tissue (CADUCEUS study). The cells have been injected into the coronary circulation of a small number of patients with ischemic cardiomyopathy or acute MI [89, 90]. These trials first indicated the safety of the CPCs administration procedure. Initial results, obtained 6 months after injection, reported a reduction in the myocardial scar mass, although an improvement in cardiac function was only reported in the SCIPIO trial (but concerns regarding scientific integrity of the latter study have been recently raised [91]). At 1-year follow-up, the CADUCEUS study confirmed the early findings, showing decreased scar size, increased viable myocardium, and improved regional function of the infarcted myocardium [92].

#### 3.1.3. Autologous or Allogeneic Cells?

The use of “autologous” injected cells (i.e., cells isolated from the patient itself and reinjected) was first recommended to avoid the immunological problems of rejection. However, their use is limited by the fact that they are not immediately available in high number and that their isolation could be difficult in critically affected patients. Furthermore, their immunogenicity is higher than expected. Indeed, their isolation and reinjection, their long-term culture in several culture media (for the mesenchymal stem cells isolated from the bone marrow, see review [93]), and their genetic modification or their epigenetic reprogramming (for the iPSCs see [94, 95]) can increase the expression on their cell surfaces of the major histocompatibility complex (MHC in animals or HLA in humans) classes I or II antigens. These “autologous” cells could be thus rejected after their reinjection and this could explain why in human hearts the long-term survival of injected BMCs is very low: only 2–5% of the injected autologous BMCs are still present in the heart a few hours after administration [96], and among these surviving cells, only a few actually become correctly integrated cardiomyocytes. Thus, the amount of injected cells which will eventually integrate into the tissue is not sufficient to improve cardiac function. These results highlight the “paracrine” activity of the injected cells which clearly stimulates the “endogenous” cardiac cells, promotes their proliferation and differentiation, or stimulates other repair mechanisms, such as angiogenesis [97, 98].

Thus, if the injected cells can survive long enough to secrete factors able to stimulate the “endogenous” capacity of the heart to regenerate, allogeneic cell therapy can also be considered as a valid option to induce cardiac regeneration. Therefore, injection of allogeneic MSCs in infarcted rat hearts [99, 100], dog hearts [101], or pig hearts [102] is safe and improves heart function as well as injection of “autologous” MSCs. Interestingly, human cardioblasts originating from the differentiation of allogeneic MSCs were transplanted into a patient developing a cardiomyopathy and demonstrated positive therapeutical effect for more than 2 years [103]. This is also true for cardiosphere-derived cells (CDCs). Indeed, the efficiency of “allogeneic” CDC injection in patients after myocardial infarction is being evaluated in the ALLSTAR trial and will be compared to this obtained by injection of “autologous” CDCs (evaluated in the CADUCEUS trial [90, 104]). Preclinical results obtained in rats and pigs suggested that injection of allogeneic CDCs was safe, induced no immunological reaction, and acted via the same mechanisms than the autologous CDC injection [105].

The injection of allogeneic cells presents several advantages: these cells could be isolated from “healthy” donors, stocked in “biobanks” in large number, thus immediately available in high numbers for patients. However, the immunogenicity of these cells remains a major hurdle to their use in regenerative medicine. Indeed, if the human precursor cells (especially the MSCs) and the human embryonic stem cells express constitutively the HLA proteins at low levels, when stimulated with interferon gamma or fibroblast growth factor 2 (FGF-2), both cell types increased HLA protein expressions, which render these cells able to be rejected rapidly on transplantation [106–110]. That is why currently many researches are aimed at understanding how blunting host immune responses to injected cells. This concerns the development of strategies limiting, for example, the host immune response (by immunosuppressive drugs, by tolerogenic cell therapies, or also by injection of monoclonal antibodies neutralizing the host immune cells). The immunogenicity of the injected cells can also be modified by modulating the site of injection or the way of cell delivering (some biomaterials can escape from host immune reactivity) (for review see [111, 112]).

#### 3.1.4. Important Conclusions Drawn from Cellular Therapies

To sum up, clinical trials evaluating cellular therapies based on “cardiomyocyte precursor cells” from various sources have not been as successful as expected to repair the injured heart. As a matter of fact, all stem cells used in cell therapies, such as BMCs, mesenchymal or adipose tissue-derived stem cells, display important cytokine secretion. This “paracrine” activity of the injected cells seems to be responsible for the positive effects observed in injured hearts after cell injections. Indeed, secreted factors stimulate the “endogenous” cardiac cells and thus promote their proliferation, differentiation, or other repair mechanisms, such as angiogenesis [97, 98]. Thus, the differentiation of the injected cells into functional cardiomyocytes integrated to the injured hearts seems to contribute only minimally to heart regeneration.

Improving the yield of incorporation/differentiation of injected cells and stimulating growth of endogenous cardiac cells to promote heart regeneration open the way to a new therapeutic paradigm based on a pharmacological standpoint. The fact that spontaneous differentiation (although at very low rate) of endogenous CPCs occurs during life demonstrates that these cells are functional but need to be stimulated [6, 7]. Future regenerative therapies should therefore capitalize on this feature and propose novel pharmacological strategies to stimulate the proliferation and differentiation of endogenous precursor cells.

### 3.2. Pharmacological Therapies to Promote Cell Regeneration

#### 3.2.1. The Complex Micorenvironment of Niches Containing CPCs

CPCs have been shown to be more abundant in the atria, in the heart's apex, and in the epicardium [113, 114], where they are located within specialized microdomains termed niches. The niches also contain differentiated cells, such as cardiomyocytes, fibroblasts, or telocytes, which control the activation state of the CPCs via physical interactions (through cell surface receptor and adhesion molecules such as Notch-1 and integrins) or via chemical, paracrine activity (such as the secretion of cytokines and growth factors) [115].

Whereas at the resting state, CPCs in the niches are kept undifferentiated and quiescent, they become activated to proliferate and differentiate into vascular cells or cardiomyocytes following myocardial injury, especially myocardial infarction. In such conditions, the hypoxic microenvironment, as well as molecules released by dying cardiomyocytes, for example, HMGB-1, plays key roles in the activation of CPCs [39, 116, 117]. Furthermore, growing evidence also indicates that infiltrating inflammatory cells recruited within the infarcted hearts promote CPC activation within the niches by releasing a wealth of factors, including growth factors (e.g., FGF-2, VEGF), prostaglandins, and cytokines (e.g., IL-10) [49, 51, 71, 76, 118] (see also Section 2.3).

#### 3.2.2. Paracrine Activation of CPCs after Myocardial Infarction: A Role for the Brain Natriuretic Peptide?

Thus, it appears evident for us that the identification of a factor able to increase the proliferation and differentiation of the “endogenous” cardiac precursor cells could be a key point in the development of cellular therapies aimed to regenerate injured hearts. That is why we are interested in the brain natriuretic peptide (BNP).

BNP is a cardiac hormone which belongs to the natriuretic peptide family, the other members of which include the atrial natriuretic peptide (ANP) secreted by the cardiac atria and the C-type related natriuretic peptide (CNP) secreted by the brain, bone, and vascular endothelial cells. BNP was first discovered in the bovine brain but it is now well established that the main source of BNP in the body is the heart, especially the ventricles [119]. BNP binds to two distinct guanylyl cyclase receptors, denoted NPR-A and NPR-B, promoting the intracellular generation of cyclic GMP (cGMP) [120]. The accumulation of cGMP in the cytoplasm activates protein kinase G (PKG) and the phosphodiesterases 2, 3, and 5 to elicit downstream signaling [120].

#### 3.2.3. BNP Biosynthesis and Secretion

BNP is a polypeptide of 32 amino acids (32 aa) in humans and pigs and 45 aa in mice and rats. It is processed from a preprohormone of 132-aa, posttranslationally modified into a 108-aa prohormone termed proBNP. The latter is enzymatically cleaved by two convertases, namely, corin and/or furin, resulting in an inactive 76-residue amino-terminal fragment (NT-proBNP) and an active 32-aa C-terminal fragment (BNP). Plasma BNP and NT-proBNP can be detected in healthy people, as well as uncleaved proBNP and O-glycosylated proBNP, which are both biologically inactive [121]. Plasma BNP levels increase in patients with various forms of heart failure and are therefore used as a helpful clinical biomarker for the diagnosis and follow-up of cardiac dysfunction [122]. It is here important to mention that recent studies indicated that plasma BNP measured during chronic heart failure rather consists of the biologically inactive forms proBNP and O-glycosylated proBNP [121, 123, 124]. These results have raised the interesting question that heart failure might be in fact associated with a deficit of biologically active BNP [124].

**Figure 3:**
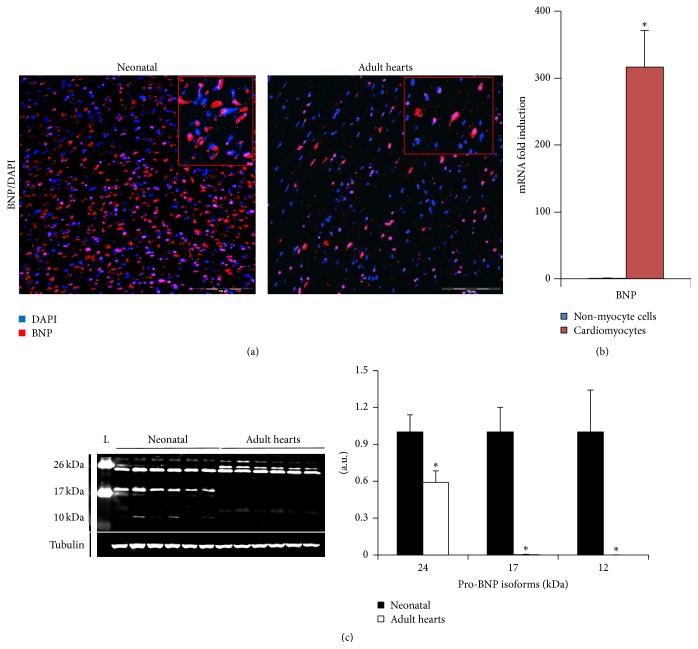
Heart expression of BNP is age-dependent and cell specific. (a) Representative microscopy pictures of neonatal and adult hearts stained for BNP (in red) and DAPI (nuclei in blue). High magnification of positive cells in top right inserts. The scale bars represent 100 *μ*m. (b) mRNA expression of BNP using quantitative PCR, in non-myocyte cells (NMCs) (blue) and cardiomyocytes (red). Results expressed as fold-increase above the levels in NMCs. *n* = 7 cardiomyocyte samples compared to 9 NMC samples. Data are means ± SEM, ^*∗*^ *P* < 0.05. (c) Determination of BNP protein levels in neonatal and adult hearts by western blot analysis. BNP protein expression with representative western blot and quantification relative to tubulin, expressed as fold changes relative to the average level of neonatal hearts. Data are means ± SEM, ^*∗*^ *P* < 0.05. a.u.: arbitrary unit.

BNP is primarily secreted by ventricular cardiomyocytes upon excessive stretch, increased transmural pressure, or direct injury (see also Figure 3(b) in neonatal hearts). Cardiac fibroblasts and endothelial cells can also secrete BNP, and, following MI, infiltrating immune cells (including neutrophils, T cells, and macrophages) may represent an additional source of BNP [125]. Interestingly, immature cells such as embryonic stem cells or also satellite cells are also able to secrete BNP [126, 127].

#### 3.2.4. Role of BNP in the Heart

Whereas the effects of BNP on the regulation of natriuresis, diuresis, and vascular tone are well documented, there remains an important gap of knowledge regarding the proper actions of BNP on the heart itself [119, 128]. In the adult, the rapid release of BNP by the heart might represent an important compensatory protective mechanism in various cardiac pathologies. In support of this assumption, it has been reported that treatment with exogenous BNP facilitated the recovery of cardiac function and improved preservation of cardiac tissue in animal models of MI. Possible mechanisms included the inhibition of cardiomyocyte apoptosis, as well as reduction of hypertrophy and fibrosis [129–134]. BNP may also modulate the immune response to cardiac injury and thereby serve to avert excessive or deregulated inflammation in this setting. Several studies performed* in vitro* indicated that BNP can inhibit monocyte chemotaxis [135], deplete the number of monocytes, B lymphocytes, and NK cells in cultured human peripheral blood mononuclear cells [136], and regulate the production of a wealth of inflammatory molecules by human macrophages [137, 138].* In vivo*, a study using transgenic mice overexpressing BNP reported increased cardiac neutrophil infiltration and MMP-9 expression after MI in transgenic animals, pointing to a key role of BNP in the processes of matrix remodeling and wound healing in this setting [138].

Several studies also pointed out a role of BNP in cardiac embryogenesis. High levels of BNP are measured during midgestation in embryonic hearts, and peaks of BNP secretion correlate with several important steps of cardiac development [139, 140]. In addition, recent findings have indicated that cardiomyocyte proliferation can be modulated during development by ANP or BNP [141]. Furthermore, it is noticeable that plasma BNP in humans is high at birth, progressively declining thereafter, to stabilize at around ten years of age to the levels found in adults [142, 143]. Taken together, these observations suggest that BNP may play important functions as a regulator of cardiomyocyte differentiation and proliferation in the developing embryo. In line with this hypothesis, it has been reported that embryonic stem cells express high levels of BNP which are essential for their proliferation and differentiation [126].

These results raise the possibility that BNP might also be involved in the process of cellular regeneration in the adult. A role of BNP was indeed reported by Kuhn et al. in the process of angiogenesis following skeletal muscle ischemia [127]. In this study, secretion of BNP by vascular satellite cells was found to activate, in a paracrine manner, the regeneration of the adjacent endothelium. So, what about cardiac regeneration?

#### 3.2.5. Role of BNP in Cardiac Regeneration

We addressed the role of BNP in cardiac regeneration in our laboratory by performing a series of experiments evaluating the relationships between CPCs and BNP both* in vitro* and* in vivo*. The first clue for an involvement of BNP in CPC proliferation and differentiation comes from our data indicating the age-dependence of BNP expression in the heart. As shown in Figure 3(a) and already published [117], more cardiac cells stained positive for BNP in mouse neonatal compared to adult hearts: in the neonatal hearts 65 ± 4% of the cardiac cells were positive for the BNP's staining compared to the adult hearts (41 ± 1% of the cells) (Figure 3(a)). BNP staining is localized around the nucleus in neonatal and adult cardiac cells (inserts Figure 3(a)). By western blot analysis, several isoforms were detected as our antibody is able to recognize all proBNP isoforms as well as the active form of the BNP (C-terminal peptide). The high molecular weight forms (24 and 17 kDa) correspond to the glycosylated proBNP isoform (Figure 3(c)) as previously described [144, 145], whereas the proBNP (12-13 kDa) was only detected in the neonatal hearts. According to previous reports, the active BNP form (10 kDa) is not detectable in neonatal or adult hearts by western blot analysis [121, 144]. All proBNP isoforms were more abundant in neonatal than in adult hearts (see quantification in Figure 3(c)).

In the neonatal hearts, BNP mRNA is 300-fold more abundant in the cardiomyocytes than in the non-myocyte cells (NMCs), suggesting that the mean source of BNP in the neonatal hearts is the cardiomyocytes (Figure 3(b)). This is also true in the adult hearts: BNP mRNA expressed by the NMCs represented less than 0.05% of the mRNA coding for BNP detected in the adult hearts (data not shown).

Further indications for a role of BNP in cardiac growth and/or regeneration come from our finding that CPCs identified* in vivo* in neonatal and adult hearts express the BNP receptors, NPR-A, and/or NPR-B [117]. Although BNP can share these receptors with other members of the natriuretic peptide family (NPR-A can also bind the atrial natriuretic peptide and NPR-B the C-type related natriuretic peptide) [120], these data strongly support that CPCs are able to respond to BNP. We then found that treatment with exogenous BNP increased the number of newly formed cardiomyocytes and of proliferating CPCs in neonatal and adult unmanipulated mice. Our next finding was that BNP injection in mice exposed to MI resulted in an increased number of CPCs and of cardiomyocytes expressing Nkx2.5, and this was associated with reduced cardiac remodeling and improved contractile function after MI [117]. Overall, our findings provided strong evidence in support of a crucial role for BNP in controlling proliferation and differentiation of CPCs after birth, therefore suggesting that the administration of BNP might be a useful therapeutic approach to promoting regeneration of the infarcted heart [117].

**Figure 4:**
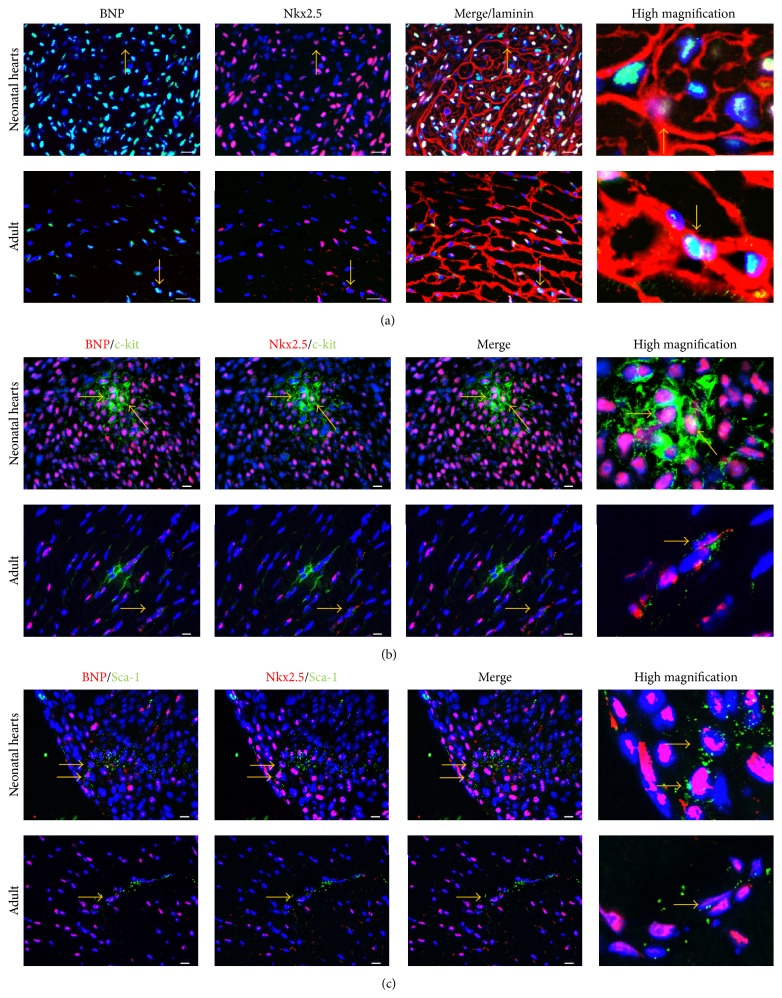
Cardiac precursor cells express BNP in neonatal and adult murine hearts. Cardiac precursor cells were defined as small Nkx2.5^+^ cells or c-kit^+^/Nkx2.5^+^ cells or Sca-1^+^/Nkx2.5^+^cells. Photomicrographs of neonatal or adult heart sections stained for BNP and DAPI (Nuclei) associated with staining for either Nkx2.5 and laminin (a), Nkx2.5 and c-kit (b), or Nkx2.5 and Sca-1 (c). Scale bars represent 80 *μ*m for the pictures in (a) and 10 *μ*m for the pictures in (b) and (c). Yellow arrows depict cells which are considered as being CPCs expressing BNP.

Furthermore, we observed also that CPCs (identified as being small laminin positive cells expressing Nkx2.5 (Nkx2.5^+^ cells) or Sca-1^+^/Nkx2.5^+^ cells or c-kit^+^/Nkx2.5^+^ cells) stained also positive for BNP, suggesting that CPCs are also able to synthesize BNP (Figure 4). CPCs could thus secrete BNP in an autocrine manner to control their proliferation and differentiation into cardiomyocytes.

#### 3.2.6. Mechanisms of BNP Actions in the Heart: Studies in NPR-A KO and NPR-B Deficient Mice

**Figure 5:**
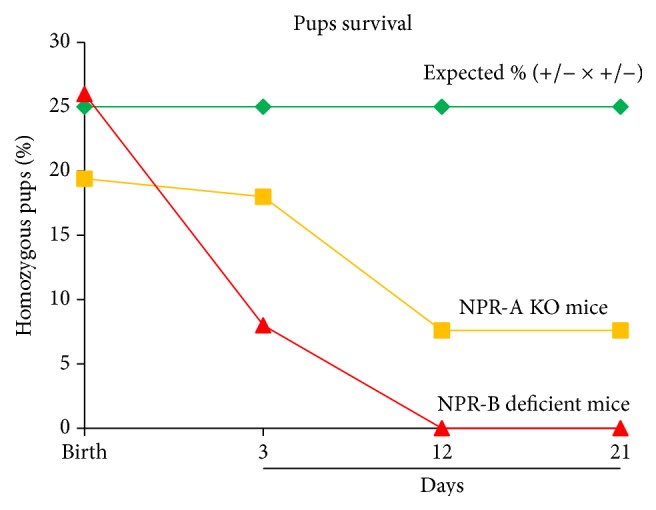
Survival curves of NPR-A KO and NPR-B deficient pups. The results are represented as percentages of the total number of pups (*n* = 66 for NPR-A KO and 96 for NPR-B deficient mice) obtained in heterozygous breeding (+/−  × +/−) and compared to the expected percentages (25%).

The demonstration that BNP has potent effects on CPCs prompted us to search for the cellular BNP receptor implicated in such actions. It is known that BNP can bind to two receptors, namely, NPR-A and NPR-B, and we therefore undertook a series of experiments using mice deficient for one or the other of these receptors [120]. We first noticed that the percentage of NPR-A KO mice at birth was lower than expected from the Mendelian frequency (19% instead of 25%), suggesting that NPR-A KO embryos die during embryogenesis, as already reported by others [146]. Furthermore, a high rate of mortality occurs in NPR-A KO pups between day 1 and day 10 (at day 10, only 8% of the surviving pups are NPR-A KO mice, instead of the expected 25%, Figure 5). In contrast, NPR-B-deficient pups are born at the expected Mendelian frequency but die within 3 days after birth (Figure 5). These observations implicate BNP receptors in biological processes critical to survival during embryogenesis and early after natal life. This assumption would be consistent with previously reported roles of BNP and BNP receptors in embryonic stem (ES) cells, as reported by Abdelalim and Tooyama [126, 147]. These authors proposed that NPR-A contributed to the self-renewal and maintenance of pluripotency of ES cells, whereas NPR-B was instead involved in their proliferation [126, 147].

Cardiac defects could be the cause of the premature death of NPR-A KO pups. Indeed, at 15.5 days of gestation, NPR-A KO embryos display a cardiomegaly without fibrosis, as well as dysregulated expression of the Cx43 protein, which could affect cardiac contractility [146]. At adulthood, NPR-A KO mice develop salt-resistant hypertension together with cardiac hypertrophy, which is out of proportion with respect to the increase in blood pressure, implying direct antihypertrophic actions of NPR-A in the heart [146, 148–150]. Concerning the NPR-B system, previous reports indicated impaired endochondral ossification, gastrointestinal tract disorders, and defects of the reproductive organs in NPR-B-deficient mice [151–155], but there is no result on their cardiac phenotype. Some information has been obtained by the use of transgenic rats expressing a dominant-negative mutant of NPR-B, which display a progressive, blood pressure-independent cardiac hypertrophy, which is further enhanced following the induction of congestive heart failure by volume overload [156]. Therefore, these results support that NPR-B is also involved in the control of cardiac growth.

**Figure 6:**
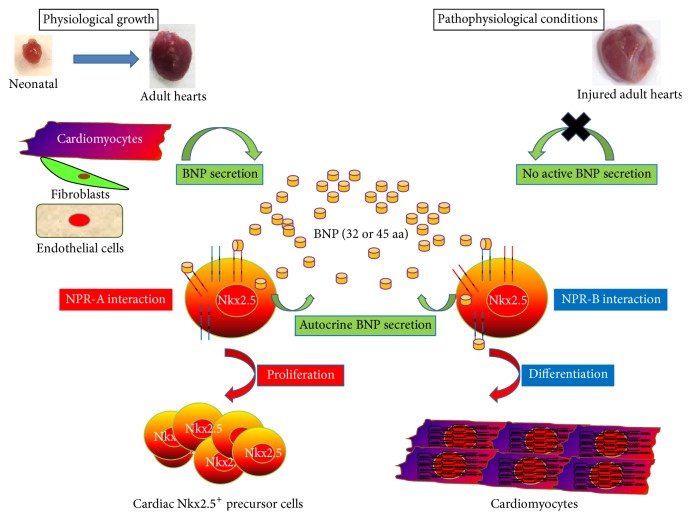
BNP modulation of cardiac precursor cell (CPC) proliferation and differentiation. BNP is secreted during physiological growth by cardiomyocytes, fibroblasts, and endothelial cells. CPCs can also secrete BNP. BNP stimulates via NPR-A CPC proliferation and via NPR-B CPC differentiation into cardiomyocytes. In pathophysiological conditions, it seems that the secreted BNP is devoid of biological activity, suggesting that BNP can be injected to stimulate the CPC proliferation and differentiation. This representation is based on the results previously published [117].

In our own studies, we recently demonstrated that both receptors control the fate of the cardiac precursor cells* in vitro* [117]. First, we demonstrated that CPCs exist in neonatal hearts of NPR-A KO and NPR-B-deficient mice. Secondly, we showed that BNP stimulates CPC proliferation* in vitro* via its binding to NPR-A (Figure 6). Thirdly, we established that BNP stimulates CPC differentiation into cardiomyocytes via binding to NPR-B in cell culture. Whether a defect in the proliferation and/or differentiation of CPCs contributes to cardiac defects and premature death in NPR-A KO and NPR-B-deficient mice remains now to be explored.

#### 3.2.7. The Use of BNP in the Clinic

The first clinical trials with recombinant human BNP (Nesiritide) in patients with acute heart failure reported positive hemodynamic and clinical effects, leading to the common use of this drug in the therapeutic arsenal of both acute and chronic heart failure. Later studies, however, raised several safety concerns about Nesiritide, the drug being possibly associated with greater risk of renal failure and higher mortality, which resulted in significant reduction in its clinical use [157, 158]. Nevertheless, more recent clinical studies reported that low doses of Nesiritide, in particular when administered via subcutaneous route, induced hemodynamic and clinical improvements without increasing nephrotoxicity or the rate of death, thus reopening the debate about the usefulness of BNP therapy in patients with heart failure [157, 159–162]. In addition, a recent meta-analysis on the use of natriuretic peptides (ANP/BNP) in patients with acute MI suggested that this treatment might protect left ventricular function [163] and a large-scale randomized clinical trial (BELIEVE II) has been recently initiated to evaluate such cardioprotective effects of low dose BNP during AMI [164].

Finally, it is particularly noteworthy that an inhibitor of neprilysin has been very recently shown to promote significant benefits in patients with chronic heart failure, when compared to angiotensin-converting enzyme inhibition (PARADIGM-HF trial) [165, 166]. Neprilysin (NEP) is an endopeptidase able to degrade several factors such as the natriuretic peptides (ANP, CNP, and BNP), but also angiotensin II, bradykinin, or endothelin-1. In the heart, NEP is expressed on the membrane of endothelial cells, vascular smooth muscle cells, fibroblasts, and cardiomyocytes and treatments of rats or rabbits with NEP inhibitors increase the blood level of BNP [167, 168]. However, NEP treatment in animal and humans has also shown to increase the blood level of angiotensin II. That is why NEP inhibitors are used with inhibitors of angiotensin-converting enzyme (ACE) such as the omapatrilat or with blocker of the angiotensin receptor, such as the LCZ696. Omapatrilat has been shown in infarcted mice to increase cardiac function and to decrease the fibrosis and the cardiomyocyte hypertrophy when compared to untreated infarcted mice [169]. However, in humans, omapatrilat was associated with development of angioedema and was not approved by the Food and Drug Administration. Thus great hope focuses now on LCZ696. In infarcted rats, LCZ696 treatment decreases the myocardial fibrosis and the cardiomyocyte hypertrophy and thus increases the ejection fraction of the treated rats compared to untreated one [170].

In patients, the mechanisms of LCZ696 leading to reduced death and rehospitalization are not yet elucidated [171]. However, increasing BNP signaling appears therefore as a meaningful and helpful strategy in patients with myocardial infarction and/or heart failure. Although it is likely that systemic vasodilation and natriuresis are key mechanisms underlying the beneficial effects of natriuretic peptides in the aforementioned studies, the positive effects of BNP on cardiac regenerative processes, as highlighted in our recent work, could also play an important role, an issue which should be critically addressed in ongoing clinical and experimental studies.

## 4. Conclusion-Future Perspectives

Therapy of cardiovascular diseases represents a major public health challenge. Primary prevention, including lifestyle modification and treatment of traditional cardiovascular risk factors, together with secondary and tertiary prevention by multidrug treatment, has been the mainstay of such therapy for decades. In recent years, novel approaches based upon the regeneration of the injured heart have been developed, holding the promise not only to relieve, but also to directly repair the damaged heart. The observation that stem cells isolated from different organs retain the ability to differentiate into mature adult beating cardiomyocytes promoted strong impetus to launch a series of clinical trials evaluating the therapeutic potential of cellular regenerative therapies in cardiac diseases. Lessons learned from these studies indicated that although such approaches appeared generally safe, their efficacy remained globally limited. Factors such as the nature of the injected cells, their number, and the route and timing of their administration emerged as critical issues which will need to be addressed in future studies to improve such efficacy. Furthermore, it has become obvious that cardiac regeneration involves complex interplays between different cell subsets of both cardiac and extracardiac (blood or bone marrow) origin, which cannot be mimicked by the one and only administration of cardiac precursor cells. A potential strategy to circumvent, at least partly, the limitations of cellular regenerative therapies could rely on the stimulation of the heart's natural ability to induce its own regeneration by pharmacological approaches. Indeed, pharmacological compounds could target not only the cellular precursors but also other cells involved in the regenerative and healing process, for instance, the fibroblasts, the endothelial cells, and the infiltrating cells, such as the different monocyte subsets. Treatment with exogenous brain natriuretic peptide is an example of such strategy, as demonstrated experimentally by its ability to induce “endogenous” cardiac regeneration. Future studies should endeavor to discover novel molecules able to stimulate such genuine capacity of the heart to regenerate, which would represent an indisputable breakthrough in the fight against cardiovascular diseases.
